# Ontology Specific Alternative Splicing Changes in Alzheimer’s Disease

**DOI:** 10.3389/fgene.2022.926049

**Published:** 2022-06-14

**Authors:** Yanjun Lu, Daoyuan Yue, Jiazhao Xie, Liming Cheng, Xiong Wang

**Affiliations:** ^1^ Department of Laboratory Medicine, Tongji Hospital, Tongji Medical College, Huazhong University of Science and Technology, Wuhan, China; ^2^ Department of Pathophysiology, Key Laboratory of Ministry of Education for Neurological Disorders, School of Basic Medicine, Tongji Medical College, Huazhong University of Science and Technology, Wuhan, China

**Keywords:** alternative splicing, alzheimer’s disease, skipped exon, retained intron, gene ontology

## Abstract

Alternative splicing (AS) is a common phenomenon and correlates with aging and aging-related disorders including Alzheimer’s disease (AD). We aimed to systematically characterize AS changes in the cerebral cortex of 9-month-old APP/PS1 mice. The GSE132177 dataset was downloaded from GEO and ENA databases, aligned to the GRCm39 reference genome from ENSEMBL via STAR. Alternative 3′SS (A3SS), alternative 5′SS (A5SS), skipped exon (SE), retained intron (RI), and mutually exclusive exons (MXE) AS events were evaluated using rMATS, rmats2sashimiplot, and maser. Differential genes or transcripts were analyzed using the limma R package. Gene ontology analysis was performed with the clusterProfiler R package. A total of 60,705 raw counts of AS were identified, and 113 significant AS events were finally selected in accordance with the selection criteria: 1) average coverage >10 and 2) delta percent spliced in (ΔPSI) >0.1. SE was the most abundant AS event (61.95%), and RI was the second most abundant AS type (13.27%), followed by A3SS (12.39%), thereafter A5SS and MXE comprised of 12.39%. Interestingly, genes that experienced SE were enriched in histone acetyltransferase (HAT) complex, while genes spliced by RI were enriched in autophagy and those which experienced A3SS were enriched in methyltransferase activity revealed by GO analysis. In conclusion, we revealed ontology specific AS changes in AD. Our analysis provides novel pathological mechanisms of AD.

## Introduction

More than 50,000 genes and 140,000 transcripts for humans have been documented in the Ensemble database, indicating an average of approximately three transcripts or isoforms for each gene caused by alternative splicing (AS). AS is mediated by the spliceosome, a large macromolecular complex that coordinates and catalyzes splicing reactions (Wilkinson et al., 2020). Introns are defined by 5′ splice site (5′SS), branch point (BP), and 3′ splice site (3′SS). The small nuclear ribonucleoproteins (snRNP) U1 and U2 recognize 5′SS and BP sequence, respectively, and the intron is released in lariat form and degraded after two transesterification reactions. Finally, the adjacent two exons are joined by a phosphodiester bond (Leung et al., 2011). Generally, five basic modes of AS are classified: alternative 3′SS (A3SS), alternative 5′SS (A5SS), skipped exon (SE), retained intron (RI), and mutually exclusive exons (MXE) (Sammeth et al., 2008). AS process orchestrates the temporal and tissue-specific expression of numerous genes during development and aging (Baralle and Giudice, 2017; Bhadra et al., 2020).

Alzheimer’s disease (AD) is the top cause of dementia and is characterized by a progressive decline of cognitive functions including memory, language, and visuospatial skills (Querfurth and LaFerla, 2010). The neuropathology of AD includes extracellular β-amyloid deposition and neurofibrillary tangles formed by hyperphosphorylated tau (Ferreira and Klein, 2011). Age, environmental and genetic risk factors also contribute to AD pathogenesis. Genetic risk factors involve amyloid precursor protein, presenilin 1 (PSEN1), sortilin-related receptor (SORL1), and triggering receptor expressed on myeloid cells 2 (TREM2) (Kulkarni et al., 2021).

Accumulating transcriptomic studies revealed AS disruption in AD (Yang et al., 2021). Variants within the fourth exon of PSEN1 cause exon 4 skipping and produce a loss of function PSEN1, leading to an early onset of AD (Tysoe et al., 1998). Monti G *et al* observed an inclusion of a novel exon in SORL1 gene that encoded a truncated protein. Moreover, this novel transcript was mainly located in neuronal dendrites and was decreased in AD patients (Monti et al., 2021). Han S *et al* observed that the second exon of TREM2 was more frequently skipped in individuals having at least one low-frequency TREM2 variant, leading to an enrichment of immune-related functional pathways revealed by gene ontology (GO) analysis of differently expressed genes (DEGs) (Han et al., 2021). SE and RI events have been widely observed in AD (Li et al., 2021; Yang et al., 2021)).

In this study, we systematically characterized AS changes in the cerebral cortex of 9-month APP/PS1 mice. We revealed ontology specific AS changes in AD.

## Materials and Methods

### Dataset and Reference Genome

GSE132177 was deposited in GEO (https://www.ncbi.nlm.nih.gov/geo/query/acc.cgi?acc=GSE132177). The raw fastq files were downloaded from the ENA database (https://www.ebi.ac.uk/ena/browser/view/PRJNA546262). This dataset performed RNA sequencing of the cerebral cortex from 9-month APP/PS1 and control mice. The reference genome was obtained from the ENSEMBL database. The gtf (http://ftp.ensembl.org/pub/release-105/gtf/mus_musculus/Mus_musculus.GRCm39.105.gtf.gz), DNA fasta (http://ftp.ensembl.org/pub/release-105/fasta/mus_musculus/dna/Mus_musculus.GRCm39.dna.primary_assembly.fa.gz), and cDNA fasta (http://ftp.ensembl.org/pub/release-105/fasta/mus_musculus/cdna/Mus_musculus.GRCm39.cdna.all.fa.gz) files were downloaded for reading alignment.

### Reads Alignment

The raw fastq files were downloaded from the ENA database using Axel (v2.17.5), and the md5 numbers were checked using md5sum (v8.30). Quality control was performed using FastQC (v0.11.9) and multiqc (v1.11). Adaptors and low-quality bases were removed using trim-galore (v0.6.7) with the following parameters: *trim_galore --phred33 -q 20 --length 36 --stringency 3 --fastqc --paired --max_n 3*. The clean data were aligned to the reference genome using STAR (v 2.7.10a) (Dobin et al., 2013).

### Gene/Transcript Expression Matrix

The aligned bam files after STAR alignment were further used to generate an expression matrix on gene level with featureCounts (v2.0.1) (Liao et al., 2014). Clean fastq files from trim-galore were further used to generate an expression matrix on transcript level with salmon (v1.0.4) (Patro et al., 2017).

### Differential Expressed Gene/Transcript Analysis

The gene expression matrix and transcript expression matrix were used for differentially expressed gene/transcript analysis using limma R package (v3.50.0) (Ritchie et al., 2015). The cutoff *p* value was 0.5, and the cutoff logFC value was 1.5.

### Alternative Splicing Analysis

The aligned and sorted bam files after STAR alignment was used for AS analysis using rMATS (v4.1.2) and rmats2sashimiplot (v2.0.4) (Shen et al., 2014). A3SS, A5SS, SE, RI, and MXE events were evaluated. Significant AS events were identified with the following criteria (Wilkinson et al., 2020): average coverage >10 (Leung et al., 2011); delta percent spliced in (ΔPSI) >0.1. The AS event statistics were performed using maser (v1.12.1) and rtracklayer (v1.54.0) R packages, and the results were plotted using echarts4r (v0.4.3), maser, and ggplot2 (v3.3.5) R packages.

### Gene Ontology Enrichment Analysis

Gene ontology (GO) enrichment analysis was used to perform enrichment analysis of differentially spliced gene sets with clusterProfiler (v4.2.2) (Wu et al., 2021), and GOplot (v1.0.2) R packages (Walter et al., 2015). Significant GO terms were collected if the q value < 0.05.

## Results

### Global Summary of Alternative Splicing Events

**FIGURE 1:**
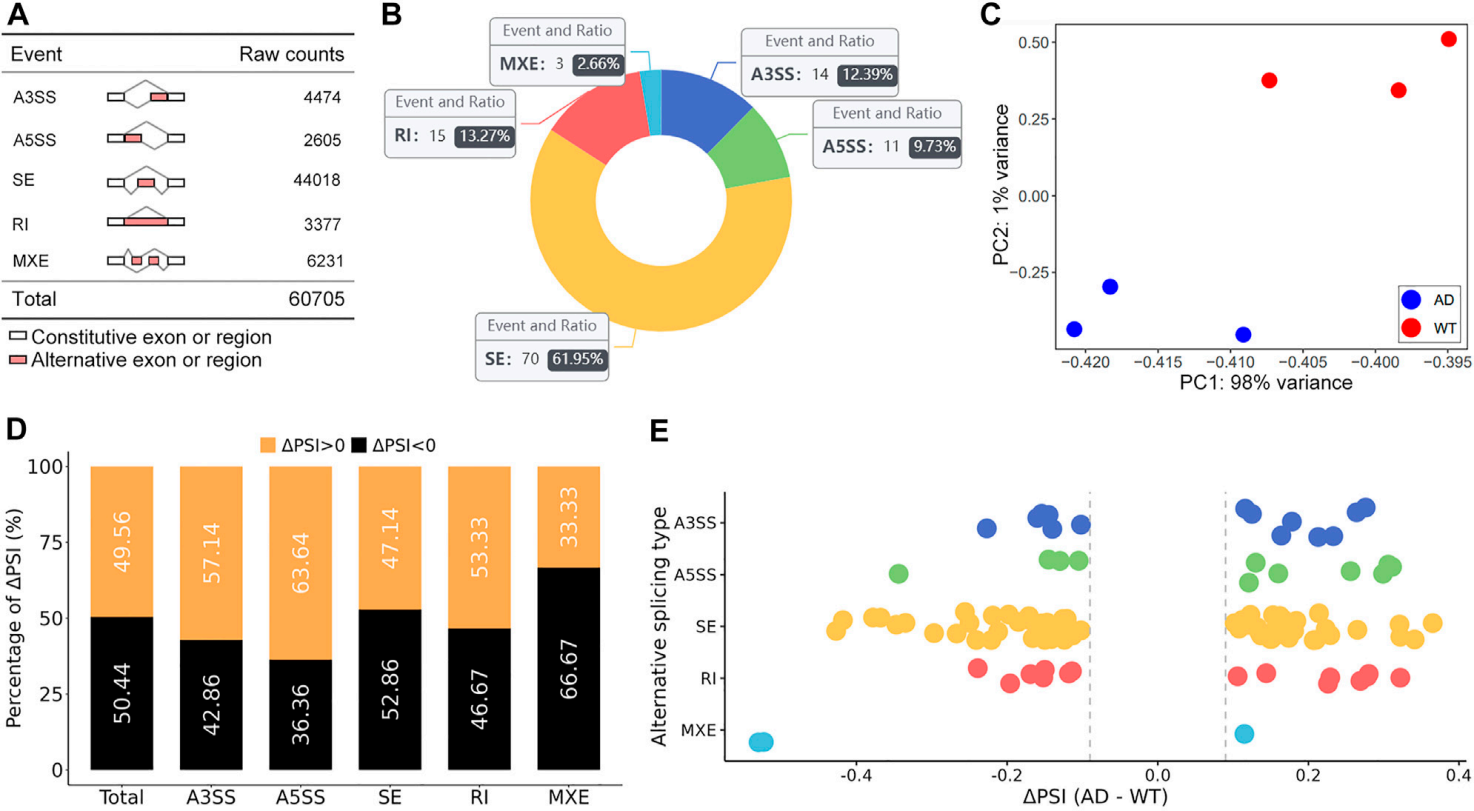
Global summary of alternative splicing events. **(A)** Summary of the raw counts in each AS event. **(B)** The number and ratio of significant AS events. **(C)** The PCA plot of the PSI values. AD and control groups were clearly classified. **(D)** The percent of ΔPSI in AS events. **(E)** The distribution of ΔPSI in AS events.

A total of 60,705 AS events were initially detected in the GSE132177 dataset (Figure 1A). Eventually, 113 significant AS events were collected according to our strict criteria (average coverage >10, ΔPSI >0.1) (Figure 1B; Table 1). The principal component analysis (PCA) of these AS events could clearly classify AD from control mice (Figure 1C). Increased PSI was found in A3SS, A5SS, and RI in the AD group, although ΔPSI showed similar changes globally (Figure 1D,E). The top three events in each type of AS pattern were included in Table 2, and all 113 significant AS events were shown in Supplementary Table S1.

**TABLE 1:** Event summary.

Event type	Total events junction counts	Average coverage >10	FDR<0.05, deltaPSI >0.1	Ratio in WT (%)	Ratio in AD (%)
A3SS	4474	2932	14	10.71	14.04
A5SS	2605	1586	11	7.14	12.28
SE	44018	34816	70	66.07	57.89
RI	3377	2046	15	12.50	14.04
MXE	6231	5189	3	3.57	1.75
Total	60705	46569	113	100	100

A3SS, alternative 3′ splice site; A5SS, alternative 5′ splice site; SE, skipped exon; RI, retained intron; MXE, mutually exclusive exons.

**TABLE 2:** Top three events in each type of AS pattern.

Id	*p* Value	FDR	ΔPSI	Gene	Type
5780	1.64E-11	7.36E-08	0.125	Rsrp1	A3SS
5943	1.95E-07	0.00029	0.276	Tmem138	A3SS
1546	5.30E-06	0.002963	0.264	Prdm16	A3SS
2080	8.34E-06	0.00345	0.299	Dab2ip	A5SS
3531	1.31E-05	0.004267	−0.145	Zfp652	A5SS
2196	2.22E-05	0.005772	−0.105	Gria3	A5SS
19005	1.23E-09	8.99E-06	0.151	Dnajc6	SE
25241	1.65E-08	4.55E-05	−0.297	1500004A13Rik	SE
31257	2.26E-08	5.85E-05	0.108	Borcs8	SE
3901	0	0	0.229	Rsrp1	RI
3018	1.07E-05	0.006047	0.278	Farsa	RI
916	1.70E-05	0.007192	0.269	Pced1a	RI
1649	4.44E-16	2.77E-12	−0.523	Tmem234	MXE
1647	4.66E-12	1.45E-08	−0.53	Tmem234	MXE
3622	9.61E-06	0.00998	0.115	Eps15l1	MXE

### SE Alternative Splicing Events

**FIGURE 2:**
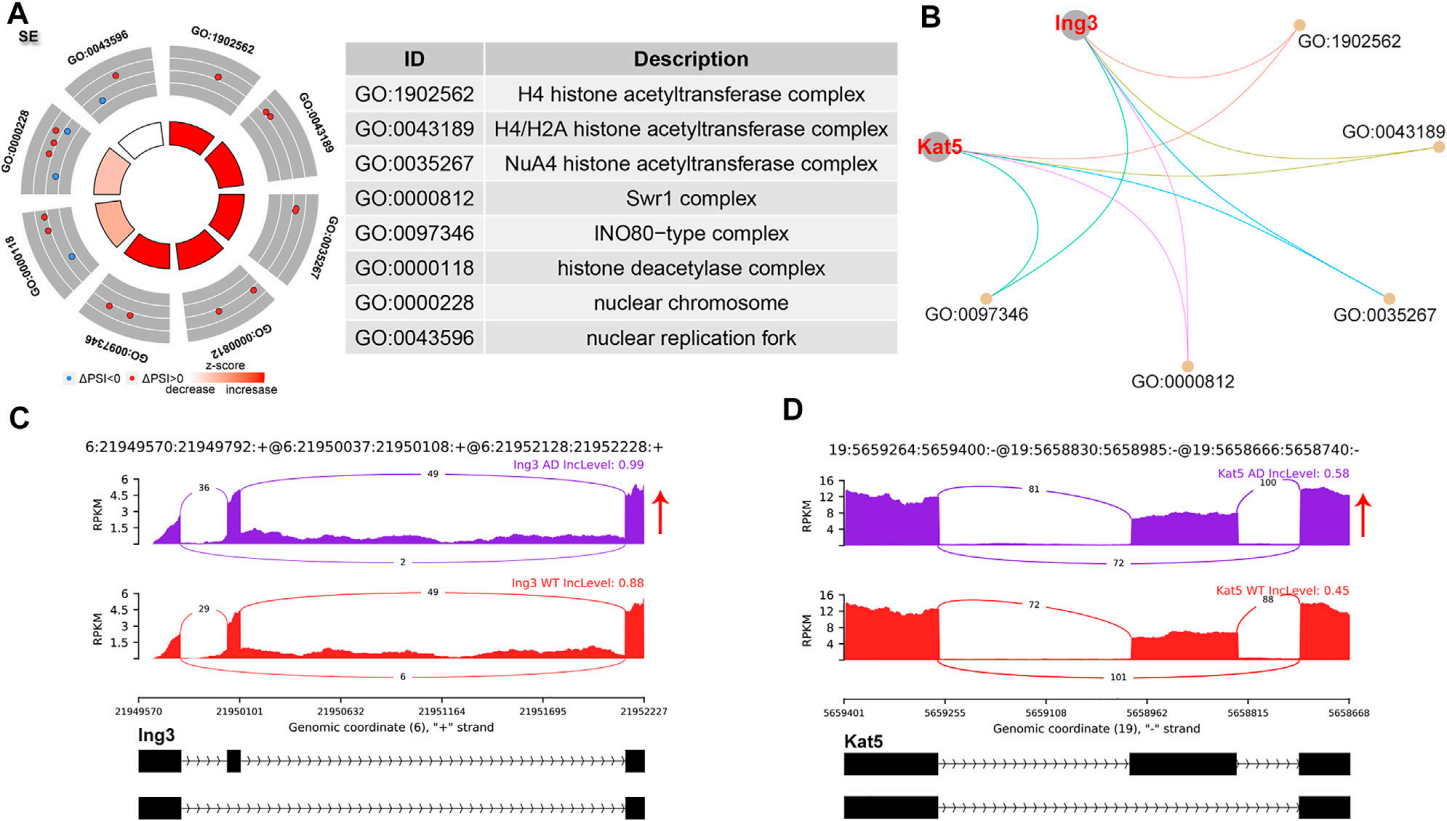
SE alternative splicing events. **(A)** Significant GO terms involving genes with significant SE events. **(B)** cnetplot showed the relation of involved genes and GO terms. Both Ing3 and Kat5 colored in red showed increased PSI level. **(C,D)** The detailed sashimi plots for Ing3 and Kat5. Red arrow represented increased PSI.

SE events were the most common AS events, accounting for 61.95% of all significant AS events (Figure 1B). GO analysis of genes with significant SE patterns were enriched in histone acetyltransferase (HAT) complex including Inhibitor of growth family member 3 (Ing3) and Lysine acetyltransferase 5 (Kat5) (Figure 2A,B). Both Ing3 and Kat5 tended to harbor longer exons in AD (Figure 2C,D).

### RI Alternative Splicing Events

**FIGURE 3:**
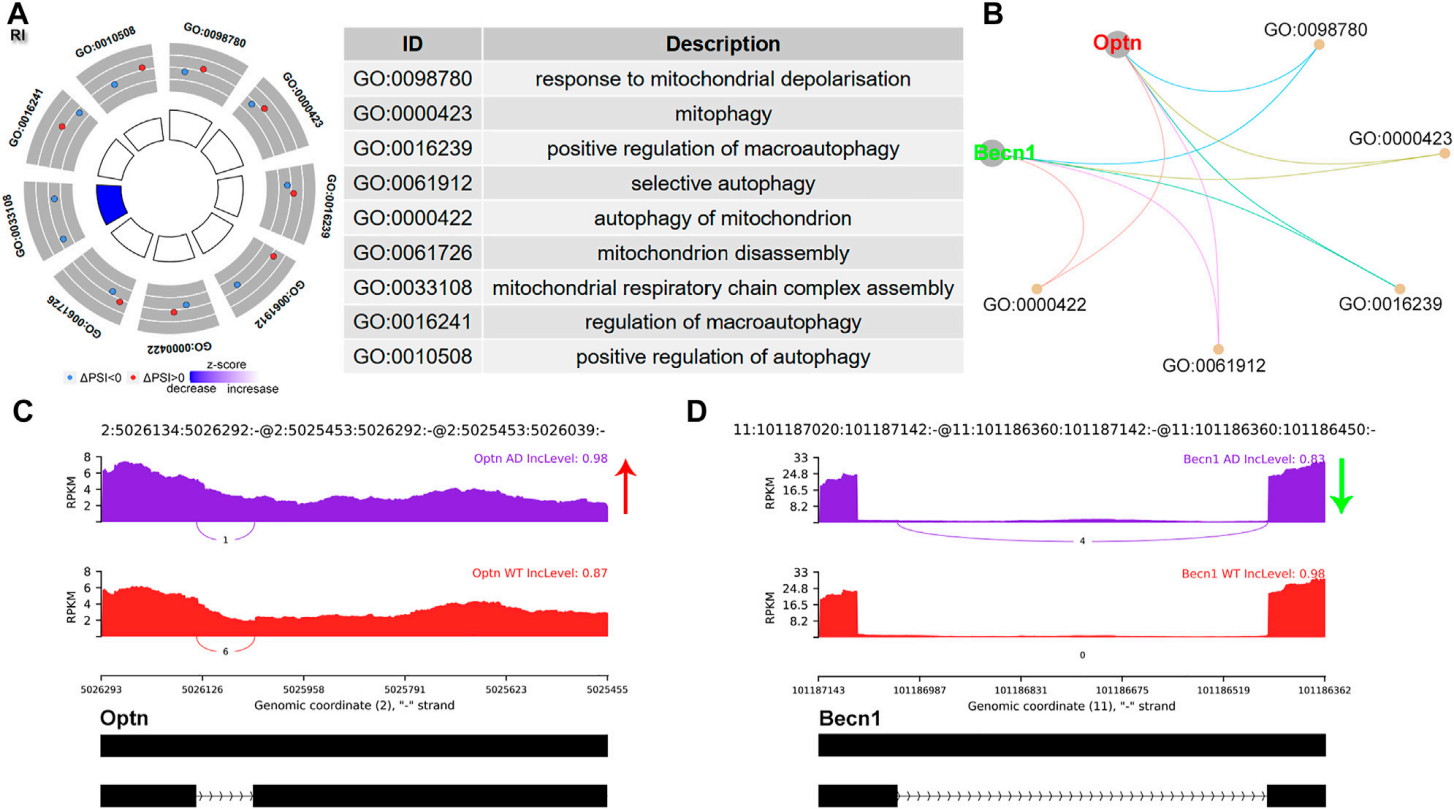
RI alternative splicing events. **(A)** Significant GO terms involving genes with significant RI events. **(B)** cnetplot showed the relation of involved genes and GO terms. Becn1 colored in green indicated decreased RI PSI levels, while Optn colored in red represented increased PSI level. **(C,D)** The detailed sashimi plots for Optn and Becn1. The red arrow represented increased PSI, while green arrow indicated decreased PSI.

RI is usually associated with decreased protein translation. The PSI referred to the inclusion level of the retained intron. A total of 15 (13.27%) significant RI events were identified. GO analysis revealed enrichment of mitochondrion autophagy, involving Optineurin (Optn) and Beclin 1 (Becn1) (Figure 3A,B). Optn showed an increased intron inclusion level (Figure 3C), while Becn1 showed decreased retained intron (Figure 3D). Becn1 plays a central role in autophagy (Wei et al., 2008).

### A3SS Alternative Splicing Events

**FIGURE 4:**
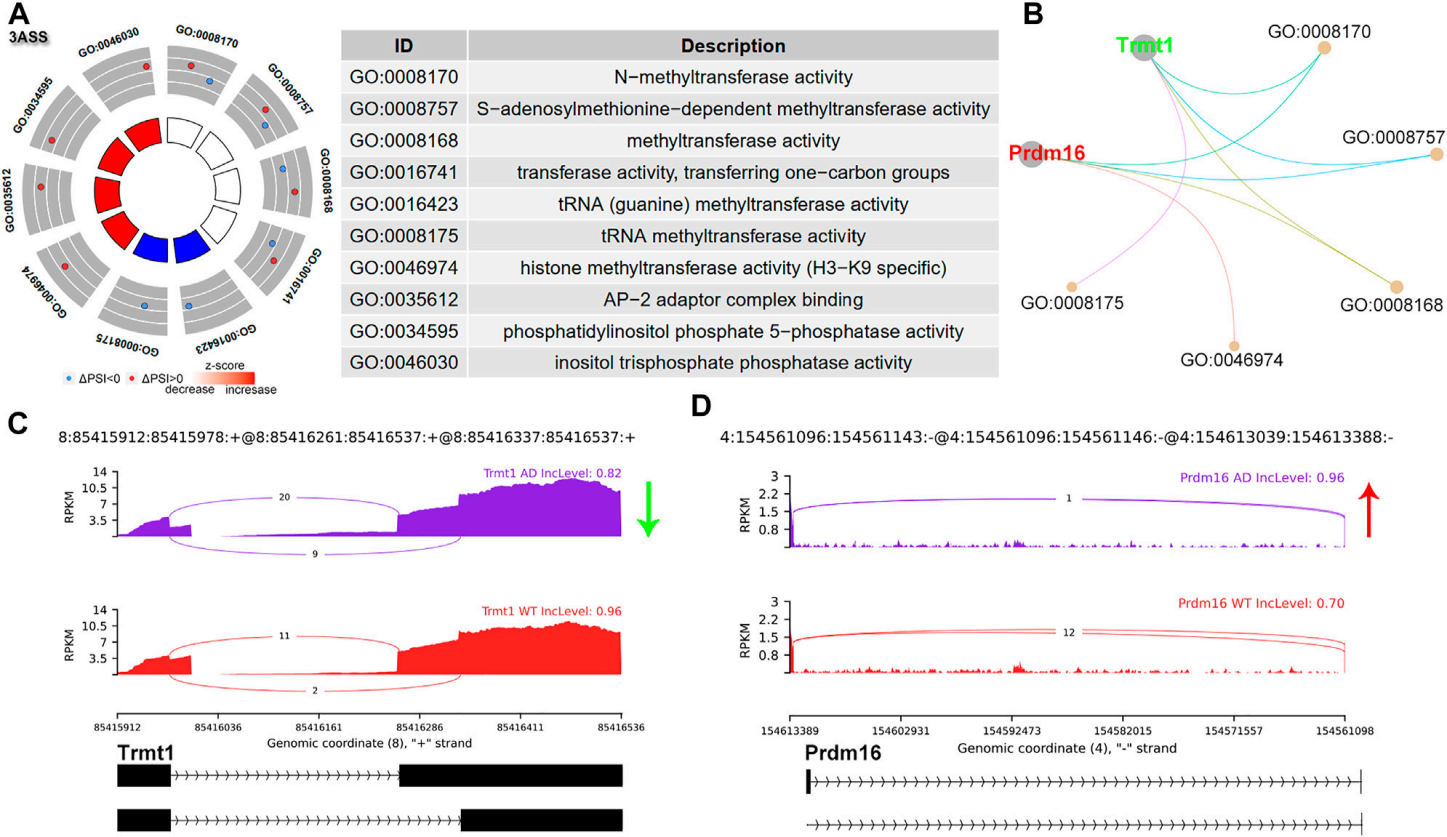
A3SS alternative splicing events. **(A)** Significant GO terms involving genes with significant A3SS events. **(B)** cnetplot showed the relation of involved genes and GO terms. Trmt1 colored in green indicated decreased A3SS PSI level, while Prdm16 colored in red represented increased PSI level. **(C,D)** The detailed sashimi plots for Trmt1 and Prdm16. The red arrow represented increased PSI, while the green arrow indicated decreased PSI.

A3SS occurred due to alternative acceptor sites, expressing shorter or longer exons. In A3SS, the PSI indicates the inclusion level for the longer exon. A total of 14 significant A3SS events were identified (Figure 1B and Supplementary Table S1). GO analysis was performed for A3SS related genes. Interestingly, genes with significant A3SS pattern were enriched in methyltransferase activity, including TRNA Methyltransferase 1 (Trmt1), and PR domain containing 16 (Prdm16) (Figure 4A,B). The sashimi plot showed decreased PSI for Trmt1, indicating a shorter exon of Trmt1 in AD (Figure 4C). Prdm16 showed increased PSI and a longer exon of Prdm16 in AD (Figure 4D). Trmt is a tRNA-modifying enzyme, which acts as a di-methyltransferase using S-adenosyl methionine as a methyl donor to modify the 26th guanine residue of the tRNA. Trmt1 was associated with neurological dysfunction (Jonkhout et al., 2021). Prdm16 displayed histone methyltransferase activity and functioned as a transcriptional regulator (Nishikata et al., 2003).

### Differentially Expressed Genes and Transcript

**FIGURE 5:**
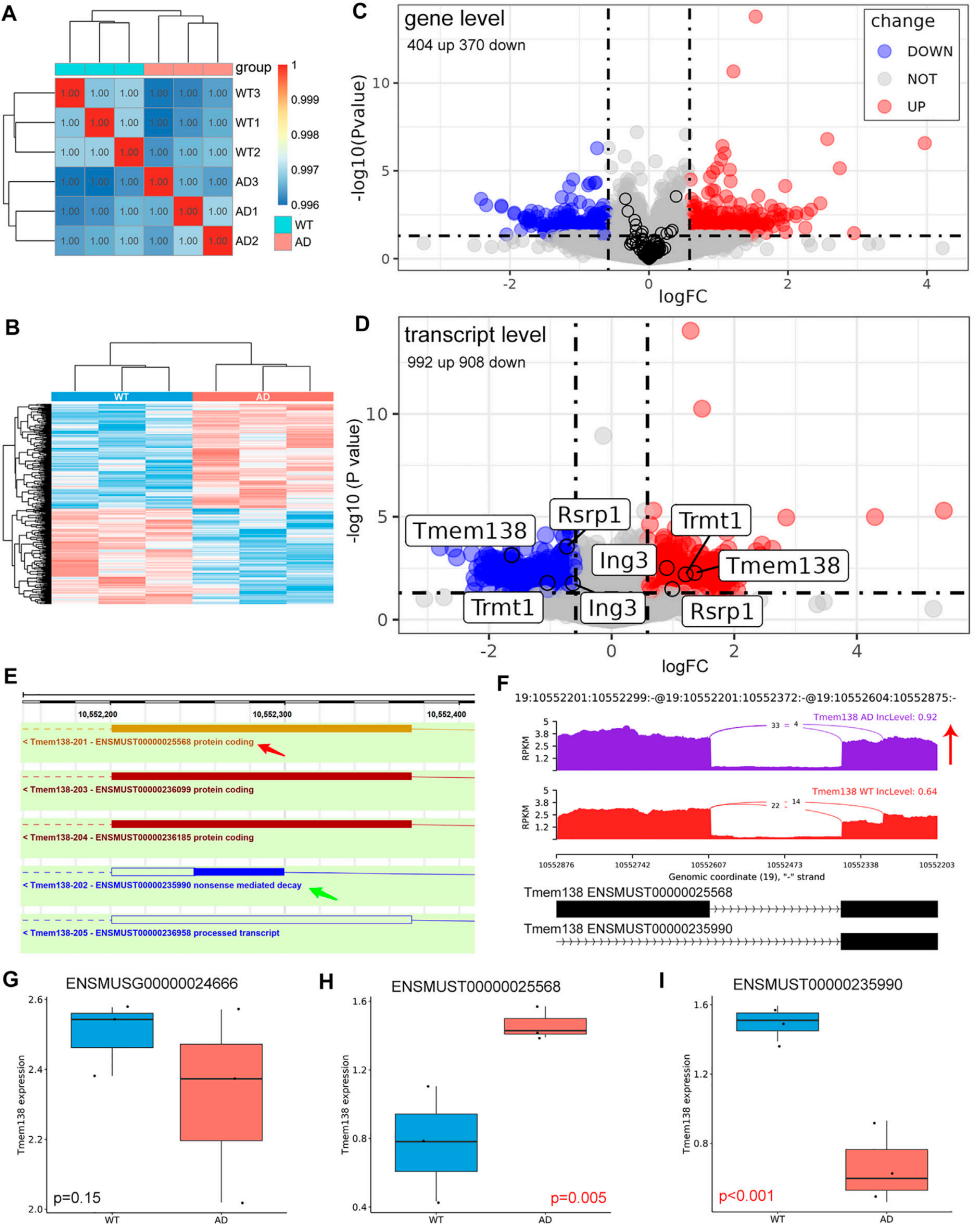
Differentially expressed genes and transcripts. **(A)** Sample correlation analysis using gene expression matrix. **(B)** Heatmap of dysregulated genes between AD and control groups. **(C)** Volcano plot of dysregulated genes. The black circle represented genes with significant AS event, and these genes did not show significant change on gene level. **(D)** Volcano plot of dysregulated transcripts. The labeled genes harbored different transcripts with opposite changes. **(E)** Genomic information of Tmem138. The red arrow indicated the longer transcript with coding ability, and the green arrow revealed the shorter transcript which may undergo NMD. **(F)** The detailed sashimi plots for Tmem138, and the red arrow represented increased PSI. **(G**–**I)** Relative expression of Tmem138 in gene and transcript levels, respectively. The longer ENSMUST00000025568 was increased while the shorter ENSMUST00000235990 was decreased in AD.

Although aberrant mRNAs arising from AS would be degraded via the nonsense-mediated mRNA decay (NMD) pathway. However, in some cases, these mRNAs may bypass NMD and be translated, especially for RI events (Forrest et al., 2004). We also examined the differentially expressed genes and transcripts in AD. These samples were well correlated with the group (Figure 5A). 404 up- and 370 down-regulated genes were identified (Figure 5B). The expression of genes with significant AS events did not show a significant difference between AD and control on gene level (Figure 5C). On transcript level, 992 up- and 908 down-regulated transcripts were identified. Several genes with significant AS events harbored transcripts with opposite changes, including Trmt1, Ing3, Rsrp1, and Tmem138 (Figure 5D; Table 3). Given Tmem138 for instance, the A3SS event resulted in a shorter transcript (ENSMUST00000235990) which underwent NMD, and a longer transcript (ENSMUST00000025568) with protein coding ability (Figure 5E). In the A3SS event, Tmem138 showed increased PSI for the longer transcript (Figure 5F). The gene level expression showed no significant difference in Tmem138 (Figure 5G). However, the longer ENSMUST00000025568 was increased while the shorter ENSMUST00000235990 was decreased in AD (Figure 5H,I), consistent with the A3SS result (Figure 5F). These results suggest that AS may alter the gene expression on transcript level rather than gene level.

**TABLE 3:** Dysregulated transcripts involved in significant AS genes.

Id	Gene	Transcript	logFC	*p* Value	Change
18692	Borcs8	ENSMUST00000123760	1.020448	0.009799	UP
15339	Borcs8	ENSMUST00000110139	1.051684	0.029002	UP
29162	Btbd8	ENSMUST00000152474	−0.79053	0.029368	DOWN
39178	Chfr	ENSMUST00000197968	0.826185	0.028575	UP
15538	Dcun1d2	ENSMUST00000110839	−1.07889	0.00516	DOWN
11548	Ddhd1	ENSMUST00000087320	−0.60375	0.002007	DOWN
14296	Dnajc6	ENSMUST00000106930	0.618619	0.00106	UP
46691	Ermard	ENSMUST00000226599	0.94063	0.016823	UP
17011	Ing3	ENSMUST00000115389	0.899232	0.003086	UP
23256	Ing3	ENSMUST00000136200	−0.63228	0.017412	DOWN
33408	Mdga1	ENSMUST00000168044	1.104151	0.034109	UP
19211	Optn	ENSMUST00000125203	−0.62723	0.033085	DOWN
37077	Pdzd2	ENSMUST00000187398	−1.40437	0.043823	DOWN
11483	Pus1	ENSMUST00000086643	1.62418	0.031663	UP
10747	Rsrp1	ENSMUST00000078084	−0.72837	0.000284	DOWN
26558	Rsrp1	ENSMUST00000145364	0.988984	0.03247	UP
40879	Sema6c	ENSMUST00000204709	−1.30559	0.023373	DOWN
34638	Siglech	ENSMUST00000173835	0.826854	0.047645	UP
6304	Sik2	ENSMUST00000041375	−0.69521	0.03334	DOWN
31551	Tle3	ENSMUST00000160882	0.981684	0.038223	UP
31491	Tle3	ENSMUST00000160724	0.727575	0.046688	UP
48977	Tmem138	ENSMUST00000235990	−1.62863	0.000732	DOWN
2774	Tmem138	ENSMUST00000025568	1.354949	0.005409	UP
21420	Tor1aip2	ENSMUST00000131359	1.211702	0.023502	UP
35047	Trmt1	ENSMUST00000175980	1.207114	0.006327	UP
35484	Trmt1	ENSMUST00000177531	−1.04671	0.016455	DOWN
27869	Zfp652	ENSMUST00000148945	0.819442	0.014392	UP

## Discussion

AS is a widespread phenomenon, generating multiple isoforms of a single gene. Here, we investigated the main five AS patterns in the cerebral cortex of 9-month APP/PS1 mice. SE was the most abundant AS event (61.95%), and RI was the second most abundant AS type (13.27%), followed by A3SS (12.39%), then A5SS and MXE comprised 12.39%.

SE is reported to be the most common and widely investigated AS event due to shifting of the open reading frame or loss of functional domains/sites, leading to numerous diseases and considered therapeutic targets, and a variety of SE events have been found in AD human and transgenic mice (Yang et al., 2021). Apolipoprotein E receptor 2 generated an isoform lacking exon 19 though SE and was associated with impairments of spatial learning and long-term memory storage in AD (Forrest et al., 2004; Hinrich et al., 2016). Antisense oligonucleotide targeting intronic splicing silencer, which increased exon 19 inclusion, improved the mentioned cognitive defects in AD mice (Hinrich et al., 2016), providing novel therapeutic strategies for AD. We observed that HAT complex-related genes were spliced via the SE pathway. 16 proteins localized to the h4 HAT complex including Ing3 and Kat5(Rouillard et al., 2016). Both Ing3 and Kat5 showed increased exon inclusion of the skipped exons, leading to the production of longer transcripts. Ing3 activates p53 trans-activated promoters and interacts with TP53 to suppress cell growth and induce apoptosis (Wu et al., 2020). Kat5 is a major histone acetyltransferase that plays important roles in the regulation of DNA repair, autophagy, proteasome-dependent protein turnover, and learning and memory. Disruption of Kat5 mediated histone acetylation was an early common event in neurodegenerative disorders including AD (Beaver et al., 2020; Li and Rasmussen, 2020).

RI is the least understood mode of AS and ranked the second most common AS event in this study. Most retained introns were degraded by the NMD pathway due to the premature termination codons in the newly formed transcript. However, some mRNAs may bypass NMD and be translated (Beaver et al., 2020; Li and Rasmussen, 2020). Li HD *et al* performed integrative functional genomic analysis of RI in AD and observed remarkable correlations of RI transcripts within innate immune genes. They further identified splicing-related genes which may regulate RI events in AD (Beaver et al., 2020; Li and Rasmussen, 2020). Here, we found that genes spliced by RI were enriched in autophagy. Becn1 is the first described mammalian autophagy gene and plays a central role in autophagy. Mitochondria selective autophagy plays a critical role in various biological processes including the elimination of the damaged mitochondria. Cheng B *et al* reported that a short Becn1 isoform, which resulted from exon skipping of exon 10–11, was indispensable for mitochondria-selective autophagy, while the full-length Becn1 was essential for nonselective macroautophagic induction (Cheng et al., 2015). Whether novel Becn1 isoforms derived from RI contribute to autophagy regulation deserves further investigation.

On the other hand, A3SS and A5SS are relatively poorly characterized, although they were found to be associated with several diseases due to aberrant splicing. During A3SS and A5SS events, exons are flanked on one constitutive splice site and are flanked by two competing splice sites on the opposite side, leading to an included or excluded alternate region within the transcript. Here we found that A3SS was enriched in methyltransferase activity. Prdm16 is a transcription factor that contains an N-terminal PR domain. Discriminative AS events of Prdm16 transcripts were noted. Exon16 skipped Prdm16 showed a stronger effect than full-length isoform on promoting the transcriptional activity of the PGC-1α promoter (Chi and Lin, 2018). These results support the diverse functions for differentially spliced isoforms.

AS may result in the expression changes of corresponding transcripts or genes. We identified 404 up- and 370 down-regulated genes. On the other hand, we identified 992 up- and 908 down-regulated transcripts. Moreover, genes with significant AS events did not show a significant change of expression on gene level (Figure 5C), however, some of these genes harbored different transcripts with opposite changes on transcript level (Figure 5D). More importantly, we found that Tmem138 showed increased A3SS of ENSMUST00000025568 transcript, which was positively correlated with the expression of this transcript. The decreased transcript of Tmem138 (ENSMUST00000235990) showed reduced transcript expression, while Tmem138 showed no significant change in gene level (Figure 5E–I). These data suggest that AS may cause expression changes on the transcript level. We have also explored the potential splicing factors (SFs) involved in AS (Sveen et al., 2011), however, we did not find any SFs with significant change on gene level (Supplementary Figure S1).

In conclusion, we revealed ontology-specific AS changes in AD. SE genes were enriched in HAT complex, RI genes were enriched in autophagy, and A3SS genes were enriched in methyltransferase activity. Our analysis provides novel pathological mechanisms of AD.

## Abbreviations

AS, alternative splicing; 5′SS, 5′ splice site; BP, branch point; 3′SS, 3′ splice site; snRNP, small nuclear ribonucleoprotein; A3SS, alternative 3′SS; A5SS, alternative 5′SS; SE, skipped exon; RI, retained intron; MXE, mutually exclusive exons; AD, Alzheimer’s disease; PSEN1, presenilin 1; SORL1, sortilin-related receptor; TREM2, and triggering receptor expressed on myeloid cells 2; PSI, percent spliced in; GO, gene ontology; DEGs, differently expressed genes; PCA, principal component analysis; HAT, histone acetyltransferase; Ing3, Inhibitor of growth family member 3; Kat5, Lysine acetyltransferase 5 (Kat5); Optn, Optineurin; Becn1, Beclin 1; Trmt1, TRNA Methyltransferase 1; Prdm16, PR domain.

## Data Availability

A publicly available dataset (GSE132177) submitted by Jun Wan and Nana Ma (https://www.ncbi.nlm.nih.gov/geo/query/acc.cgi?acc=GSE132177) was used in this study. The code of this study was written by our team and was deposited in Github (https://github.com/tjhwangxiong/AD-GSE132177-Alternative-Splicing-pipeline).

## Supplementary Material

The Supplementary Material for this article can be found online at: https://www.frontiersin.org/articles/10.3389/fgene. 2022.926049/full#supplementary-material

## References

[B1] Baralle F. E. Giudice J. (2017). Alternative Splicing as a Regulator of Development and Tissue Identity. Nat. Rev. Mol. Cell Biol. 18 (7), 437–451. Epub 2017/05/11. 10.1038/nrm.2017.27 28488700 PMC6839889

[B2] Beaver M. Bhatnagar A. Panikker P. Zhang H. Snook R. Parmar V. (2020). Disruption of Tip60 HAT Mediated Neural Histone Acetylation Homeostasis Is an Early Common Event in Neurodegenerative Diseases. Sci. Rep. 10 (1), 18265. 10.1038/s41598-020-75035-3 33106538 PMC7588445

[B3] Bhadra M. Howell P. Dutta S. Heintz C. Mair W. B. (2020). Alternative Splicing in Aging and Longevity. Hum. Genet. 139 (3), 357–369. 10.1007/s00439-019-02094-6 31834493 PMC8176884

[B4] Cheng B. Xu A. Qiao M. Wu Q. Wang W. Mei Y. (2015). BECN1s, a Short Splice Variant of BECN1, Functions in Mitophagy. Autophagy 11 (11), 2048–2056. 10.1080/15548627.2015.1100785 26649941 PMC4824595

[B5] Chi Y.-L. Lin J.-C. (2018). RBM4a Modulates the Impact of PRDM16 on Development of Brown Adipocytes through an Alternative Splicing Mechanism. Biochimica Biophysica Acta (BBA) - Mol. Cell Res. 1865 (11 Pt A), 1515–1525. 10.1016/j.bbamcr.2018.08.001 30327195

[B6] Dobin A. Davis C. A. Schlesinger F. Drenkow J. Zaleski C. Jha S. (2013). STAR: Ultrafast Universal RNA-Seq Aligner. Bioinformatics 29 (1), 15–21. 10.1093/bioinformatics/bts635 23104886 PMC3530905

[B7] Ferreira S. T. Klein W. L. (2011). The Aβ Oligomer Hypothesis for Synapse Failure and Memory Loss in Alzheimer's Disease. Neurobiol. Learn. Mem. 96 (4), 529–543. 10.1016/j.nlm.2011.08.003 21914486 PMC4390395

[B8] Forrest S. T. Barringhaus K. G. Perlegas D. Hammarskjold M.-L. McNamara C. A. (2004). Intron Retention Generates a Novel Id3 Isoform that Inhibits Vascular Lesion Formation. J. Biol. Chem. 279 (31), 32897–32903. 10.1074/jbc.M404882200 15159391

[B9] Han S. Na Y. Koh I. Nho K. Lee Y. (2021). Alternative Splicing Regulation of Low-Frequency Genetic Variants in Exon 2 of TREM2 in Alzheimer's Disease by Splicing-Based Aggregation. Ijms 22 (18), 9865. 10.3390/ijms22189865 34576031 PMC8471326

[B10] Hinrich A. J. Jodelka F. M. Chang J. L. Brutman D. Bruno A. M. Briggs C. A. (2016). Therapeutic Correction of ApoER2 Splicing in Alzheimer's Disease Mice Using Antisense Oligonucleotides. EMBO Mol. Med. 8 (4), 328–345. 10.15252/emmm.201505846 26902204 PMC4818756

[B11] Jonkhout N. Cruciani S. Santos Vieira H. G. Tran J. Liu H. Liu G. (2021). Subcellular Relocalization and Nuclear Redistribution of the RNA Methyltransferases TRMT1 and TRMT1L upon Neuronal Activation. RNA Biol. 18 (11), 1905–1919. 10.1080/15476286.2021.1881291 33499731 PMC8583002

[B12] Kulkarni B. Kumar D. Cruz-Martins N. Sellamuthu S. (2021). Role of TREM2 in Alzheimer's Disease: A Long Road Ahead. Mol. Neurobiol. 58 (10), 5239–5252. 10.1007/s12035-021-02477-9 34275100

[B13] Leung A. K. W. Nagai K. Li J. (2011). Structure of the Spliceosomal U4 snRNP Core Domain and its Implication for snRNP Biogenesis. Nature 473 (7348), 536–539. 10.1038/nature09956 21516107 PMC3103711

[B14] Li H. D. Funk C. C. McFarland K. Dammer E. B. Allen M. Carrasquillo M. M. (2021). Integrative Functional Genomic Analysis of Intron Retention in Human and Mouse Brain with Alzheimer's Disease. Alzheimer's Dementia 17 (6), 984–1004. 10.1002/alz.12254 PMC8248162 33480174

[B15] Li Z. Rasmussen L. J. (2020). TIP60 in Aging and Neurodegeneration. Ageing Res. Rev. 64, 101195. 10.1016/j.arr.2020.101195 33091598

[B16] Liao Y. Smyth G. K. Shi W. (2014). featureCounts: an Efficient General Purpose Program for Assigning Sequence Reads to Genomic Features. Bioinformatics 30 (7), 923–930. 10.1093/bioinformatics/btt656 24227677

[B17] Monti G. Kjolby M. Jensen A. M. G. Allen M. Reiche J. Møller P. L. (2021). Expression of an Alternatively Spliced Variant of SORL1 in Neuronal Dendrites Is Decreased in Patients with Alzheimer's Disease. Acta Neuropathol. Commun. 9 (1), 43. 10.1186/s40478-021-01140-7 33726851 PMC7962264

[B18] Nishikata I. Sasaki H. Iga M. Tateno Y. Imayoshi S. Asou N. (2003). A Novel EVI1 Gene Family, MEL1, Lacking a PR Domain (MEL1S) Is Expressed Mainly in T(1;3)(p36;q21)-Positive AML and Blocks G-CSF-Induced Myeloid Differentiation. Blood 102 (9), 3323–3332. 10.1182/blood-2002-12-3944 12816872

[B19] Patro R. Duggal G. Love M. I. Irizarry R. A. Kingsford C. (2017). Salmon Provides Fast and Bias-Aware Quantification of Transcript Expression. Nat. Methods 14 (4), 417–419. 10.1038/nmeth.4197 28263959 PMC5600148

[B20] Querfurth H. W. LaFerla F. M. (2010). Alzheimer's Disease. N. Engl. J. Med. 362 (4), 329–344. 10.1056/NEJMra0909142 20107219

[B21] Ritchie M. E. Phipson B. Wu D. Hu Y. Law C. W. Shi W. (2015). Limma Powers Differential Expression Analyses for RNA-Sequencing and Microarray Studies. Nucleic Acids Res. 43 (7), e47. 10.1093/nar/gkv007 25605792 PMC4402510

[B22] Rouillard A. D. Gundersen G. W. Fernandez N. F. Wang Z. Monteiro C. D. McDermott M. G. (2016). The Harmonizome: a Collection of Processed Datasets Gathered to Serve and Mine Knowledge about Genes and Proteins. Database 2016, baw100. 10.1093/database/baw100 27374120 PMC4930834

[B23] Sammeth M. Foissac S. Guigó R. (2008). A General Definition and Nomenclature for Alternative Splicing Events. PLoS Comput. Biol. 4 (8), e1000147. 10.1371/journal.pcbi.1000147 18688268 PMC2467475

[B24] Shen S. Park J. W. Lu Z.-x. Lin L. Henry M. D. Wu Y. N. (2014). rMATS: Robust and Flexible Detection of Differential Alternative Splicing from Replicate RNA-Seq Data. Proc. Natl. Acad. Sci. U.S.A. 111 (51), E5593–E5601. 10.1073/pnas.1419161111 25480548 PMC4280593

[B25] Sveen A. Ågesen T. H. Nesbakken A. Rognum T. O. Lothe R. A. Skotheim R. I. (2011). Transcriptome Instability in Colorectal Cancer Identified by Exon Microarray Analyses: Associations with Splicing Factor Expression Levels and Patient Survival. Genome Med. 3 (5), 32. 10.1186/gm248 21619627 PMC3219073

[B26] Tysoe C. Whittaker J. Xuereb J. Cairns N. J. Cruts M. Van Broeckhoven C. (1998). A Presenilin-1 Truncating Mutation Is Present in Two Cases with Autopsy-Confirmed Early-Onset Alzheimer Disease. Am. J. Hum. Genet. 62 (1), 70–76. 10.1086/301672 9443865 PMC1376799

[B27] Walter W. Sánchez-Cabo F. Ricote M. (2015). GOplot: an R Package for Visually Combining Expression Data with Functional Analysis: Fig. 1. Bioinformatics 31 (17), 2912–2914. 10.1093/bioinformatics/btv300 25964631

[B28] Wei Y. Pattingre S. Sinha S. Bassik M. Levine B. (2008). JNK1-mediated Phosphorylation of Bcl-2 Regulates Starvation-Induced Autophagy. Mol. Cell 30 (6), 678–688. 10.1016/j.molcel.2008.06.001 18570871 PMC2478643

[B29] Wilkinson M. E. Charenton C. Nagai K. (2020). RNA Splicing by the Spliceosome. Annu. Rev. Biochem. 89, 359–388. 10.1146/annurev-biochem-091719-064225 31794245

[B30] Wu T. Hu E. Xu S. Chen M. Guo P. Dai Z. (2021). clusterProfiler 4.0: A Universal Enrichment Tool for Interpreting Omics Data. Innovation 2 (3), 100141. 10.1016/j.xinn.2021.100141 34557778 PMC8454663

[B31] Wu X. Chen C. Luo B. Yan D. Yan H. Chen F. (2020). Nuclear ING3 Expression Is Correlated with a Good Prognosis of Breast Cancer. Front. Oncol. 10, 589009. 10.3389/fonc.2020.589009 33469513 PMC7813678

[B32] Yang M. Ke Y. Kim P. Zhou X. (2021). ExonSkipAD Provides the Functional Genomic Landscape of Exon Skipping Events in Alzheimer's Disease. Brief. Bioinform 22 (5). 10.1093/bib/bbaa438 PMC8425305 33497435

