# Supplementary figures and images for: Ontology Specific Alternative Splicing Changes in Alzheimer’s Disease
Source: Front Genet. 2022 Jun 14;13:926049. doi: 10.3389/fgene.2022.926049 (PMC9237535; doi:10.3389/fgene.2022.926049)

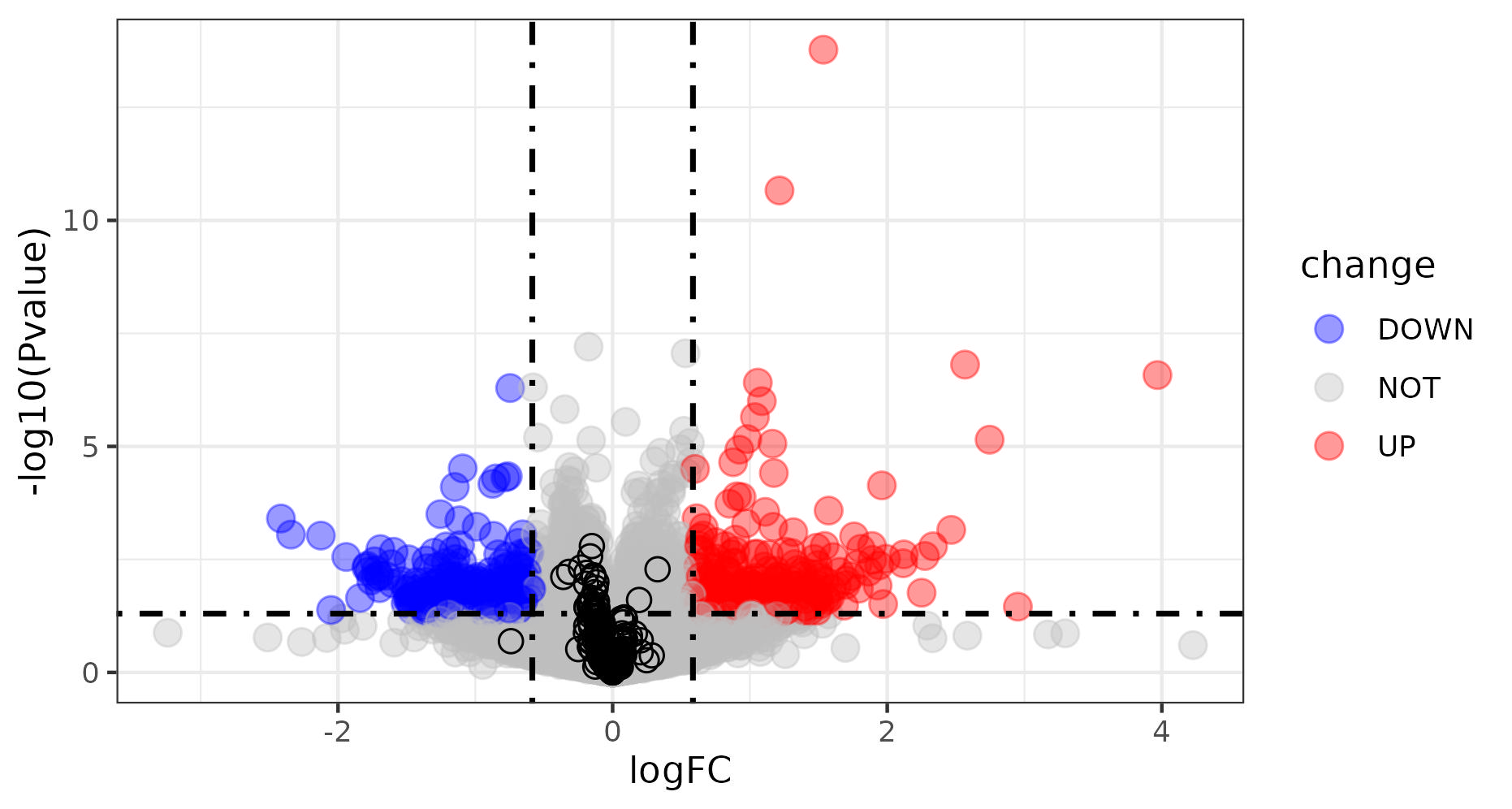

Supplement: Supplementary file 1 [file DataSheet1.ZIP › Figure S1.jpg]
